# Predictors of Mortality in Elderly and Very Elderly Emergency Patients with Sepsis: A Retrospective Study

**DOI:** 10.5811/westjem.2020.7.47405

**Published:** 2020-10-06

**Authors:** Phetsinee Boonmee, Onlak Ruangsomboon, Chok Limsuwat, Tipa Chakorn

**Affiliations:** Siriraj Hospital, Mahidol University, Department of Emergency Medicine, Bangkok, Thailand

## Abstract

**Introduction:**

Elderly patients are at increased risk of developing sepsis and its adverse outcomes. Diagnosing and prognosing sepsis is particularly challenging in older patients, especially early at emergency department (ED) arrival. We aimed to study and compare the characteristics of elderly and very elderly ED patients with sepsis and determine baseline factors associated with in-hospital mortality. We also compared prognostic accuracy of the criteria for systemic inflammatory response syndrome, quick sequential organ failure assessment (qSOFA), and the National Early Warning Score in predicting mortality.

**Methods:**

We conducted a retrospective study at the ED of Siriraj Hospital Mahidol University in Bangkok, Thailand. Patients over 18 years old who were diagnosed and treated for sepsis in the ED between August 2018–July 2019 were included. We categorized patients into non-elderly (aged <65 years), elderly (aged 65–79 years), and the very elderly (aged >80 years) groups. The primary outcome was in-hospital mortality. Baseline demographics, comorbidities, source and etiology of sepsis, including physiologic variables, were compared and analyzed to identify predictors of mortality. We calculated and compared the area under the receiver operator characteristics curves (AUROC) of early warning scores.

**Results:**

Of 1616 ED patients with sepsis, 668 (41.3%) were very elderly, 512 (31.7%) were elderly, and 436 (27.0%) were non-elderly. The mortality rate was highest in the very elderly, followed by the elderly and the non-elderly groups (32.3%, 25.8%, and 24.8%, respectively). Factors associated with mortality in the very elderly included the following: age; do-not-resuscitate (DNR) status; history of recent admission <3 months; respiratory tract infection; systolic blood pressure <100 millimeters mercury (SBP<100); oxygen saturation; and Glasgow Coma Scale (GCS) score. Factors associated with mortality in the elderly were DNR status, body temperature, and GCS score. qSOFA had the highest AUROC in predicting in-hospital mortality in both very elderly and elderly patients (AUROC 0.60 [95% confidence interval {CI}, 0.55–0.65] and 0.55 [95% CI, 0.49–0.61, respectively]).

**Conclusion:**

The mortality rate in the very elderly was higher than in the younger populations. Age, DNR status, recent admission, respiratory tract infection, SBP<100, oxygen saturation. and GCS score independently predicted hospital mortality in very elderly patients. The qSOFA score had better but only moderate accuracy in predicting mortality in elderly and very elderly sepsis patients.

## INTRODUCTION

The elderly population is increasing worldwide due to an increase in life expectancy and a decrease in birth rate. It is estimated that this population will grow the most rapidly and will surpass that of the younger population by 2050.^1^ The use of healthcare resources is thereby increasing, as more than half of patients requiring intensive care unit (ICU) admissions are elderly (aged over 65 years).^2^–^4^ As for the emergency department (ED), the mean age of ED patients is also increasing. Elderly patients have become “frequent users” of the ED.^5^–^6^

Sepsis is a state of organ dysfunction caused by dysregulated host response to infection.^7^–^8^ It is a critical condition leading to a high rate of mortality and is considered a significant health problem worldwide. The incidence of sepsis increases with age, especially in very elderly patients (age ≥80 years), and mortality is also significantly higher in this population.^9^–^10^ This high incidence and mortality could be explained by various reasons, such as multiple pre-existing comorbidities, reduced functional reserve, and abnormal immune system.^11^ Diagnosing sepsis is also more difficult, given elderly patients’ vague symptoms and atypical clinical presentations. This poses an extreme challenge for emergency physicians to recognize such patients early, especially those at greater risk of adverse outcomes.

Various diagnostic and prognostic tools have been developed and/or validated to help predict poor prognosis in suspected sepsis patients early at presentation to the ED. These tools include criteria developed especially for sepsis, such as systemic inflammatory response syndrome (SIRS),^12^ and the quick sequential organ failure assessment (qSOFA).^7^ Criteria such as the National Early Warning Score (NEWS) have been developed for other purposes but validated to predict outcomes of sepsis.^13^ These scoring systems consist of physiologic variables, such as vital signs and mental status. They have been frequently used tools to predict mortality secondary to sepsis in the ED.^14^–^16^ However, with distinctive clinical presentations in the elderly, the accuracy of these criteria may be different. To date, no studies have validated or compared these scoring systems in the ED in this specific population.

Although the mortality rate from sepsis is exceptionally high in geriatric patients, little is known about the predictive factors of this adverse outcome, especially in the very elderly group. Therefore, we conducted this study to examine the characteristics and determine factors associated with in-hospital mortality in elderly and very elderly patients who presented to the ED with sepsis. We also aimed to study the accuracy of SIRS, qSOFA, and NEWS in predicting mortality in these patients.

## METHODS

### Study Design and Setting

We conducted a retrospective study at the ED of Siriraj Hospital, Mahidol University in Bangkok, Thailand. Siriraj Hospital is the largest tertiary university hospital in Thailand, with over 20,000 ED visits per year. Siriraj Institutional Review Board approved the study (certificate of approval Si 510/2019). Patients’ informed consent was waived.

**Population Health Research Capsule**

What do we already know about this issue?

*Elderly patients are at increased risk of developing sepsis and its adverse outcomes. Diagnosing and prognosing sepsis in the elderly is particularly challenging*.

What was the research question?

*We sought to determine baseline factors associated with in-hospital mortality of elderly ED patients with sepsis*.

What was the major finding of the study?

*Age was associated with mortality only in the very elderly. qSOFA had the best prognostic utility in these patients*.

How does this improve population health?

*If the factors associated with sepsis in elderly patients are better understood, more appropriate care can be guided toward high-risk patients*.

### Patients

We assessed ED patients retrospectively and consecutively for eligibility between August 1, 2018–July 31, 2019. Adult patients aged >18 years were eligible if they were suspected of having sepsis, were treated accordingly in the ED and were discharged from the hospital with sepsis-related diagnoses (ie, sepsis, sepsis-induced hypotension, and septic shock) based on Sepsis-3.^7^ The attending emergency physicians suspected sepsis based on SIRS or qSOFA, together with clinical judgment. This suspicion of sepsis was defined by having ordered a hemoculture followed by having prescribed intravenous antibiotics, or vice versa. All patients received antibiotics within one hour after sepsis suspicion. The diagnosis of sepsis was confirmed during admission by internal medicine or ICU attending physicians. Patients with prescribed empirical antibiotics who were not considered to have sepsis and later had antibiotics ceased were excluded. After inclusion, we categorized patients by their age according to the most-often referred term into the non-elderly (aged >18 and <65 years), elderly (aged at ≥65 and <80 years), and very elderly (aged ≥80 years) patients.

### Data Variables

When patients visit the ED, they are assessed by triage nurses who record their initial vital signs in the standing triage form, before being assessed by emergency physicians. Afterward, patients’ vitals were routinely recorded every two hours. We extracted the following data from their medical records: age; gender; body temperature; heart rate; respiratory rate; blood pressure; oxygen saturation measured by pulse oximetry; mental status reported as Glasgow Coma Scale (GCS) score; baseline functional status; and comorbidities. We also collected laboratory results, management in the ED, diagnosis, disposition, outcomes, and any other relevant data. An emergency medicine resident (PM), trained by the attending emergency physician researchers (CL and OR), was the data abstractor. Another physician (OR) randomly audited the recorded data for its completeness and reliability. Interobserver agreement measured by weighted kappa on mortality status and early warning score values were 1.0 and 0.98, respectively. Respiratory rate ≥22 breaths per minute and systolic blood pressure ≤100 milligrams mercury (SBP≤100) were cut-points chosen to be analyzed according to qSOFA. Infection was deemed to be hospital-associated if patients had been admitted within the prior three months, or healthcare-associated if patients were in healthcare facilities. Otherwise, they were considered to be community acquired. The primary outcome was in-hospital mortality. For scoring systems calculation, we imputed components of each risk score from the standing ED admission triage form recorded at the patient’s ED arrival or records closest to the time that sepsis was suspected, defined as the time of culture or antibiotics, whichever came first.

SIRS is a four-item score (0–4 points) consisting of pulse rate, respiratory rate, body temperature. and white blood cell count. qSOFA contains three items (0–3 points): respiratory rate; mental status; and systolic blood pressure. NEWS (0–20 points) is an aggregated, weighted scoring system based on pulse rate, respiratory rate, body temperature, systolic blood pressure, oxygen saturation, and need for oxygen supplement.

### Statistical Analysis

We reported patients’ characteristics as frequency (percentage) and compared them using chi-squared or Fisher’s exact test for categorical variables. Continuous variables were reported as mean (standard deviation) or median (interquartile range) and compared using Student’s t-test or Mann-Whitney U test, as appropriate. We compared characteristics between two groups based on patients’ ages: 1) between the elderly and the very elderly, and 2) between the very elderly and all others (aged <80 years). Univariate logistic regression analyses were performed to evaluate factors associated with hospital mortality in each age group, and results were presented as odds ratio (95% CI) and *p*-value. We only analyzed baseline variables that could be retrieved early after ED arrival because we aimed to categorize patients at high risk early after ED primary triage. Variables, which were statistically significant or considered clinically significant in the univariate analyses, were selected for the multivariate analyses. We subsequently analyzed multivariate logistic regressions in each age group. Furthermore, we performed subgroup analyses of patients without do-not-resuscitate (DNR) status to adjust for potential bias that the status might have caused. We decided to include patients with DNR status in the primary analysis because, unlike the younger population, a significant number of elderly and very elderly patients had this status. We believed that analyzing the results both before and after its stratification could help us to better understand this distinctive population. As for scoring systems, we calculated their prognostic accuracy performances and presented them as sensitivity, specificity, positive likelihood ratio (LR+), negative likelihood ration (LR−), negative predictive value (NPV), positive predictive value (PPV), and area under the curve of the receiver operator characteristics curves (AUROC). The accuracy at recommended cut-points from previous literature (SIRS ≥2, qSOFA ≥2 and NEWS ≥5) were computed and reported. We performed analyses using SPSS 18.0 (IBM Corp., Chicago, IL), and we calculated (95% CI) for sensitivity and specificity, LR+, LR−, NPV and PPV using MedCalc statistical software for Windows version 19 (MedCalc Software Ltd, Ostend, Belgium).

## RESULTS

### Characteristics of Patients

**Figure 1:**
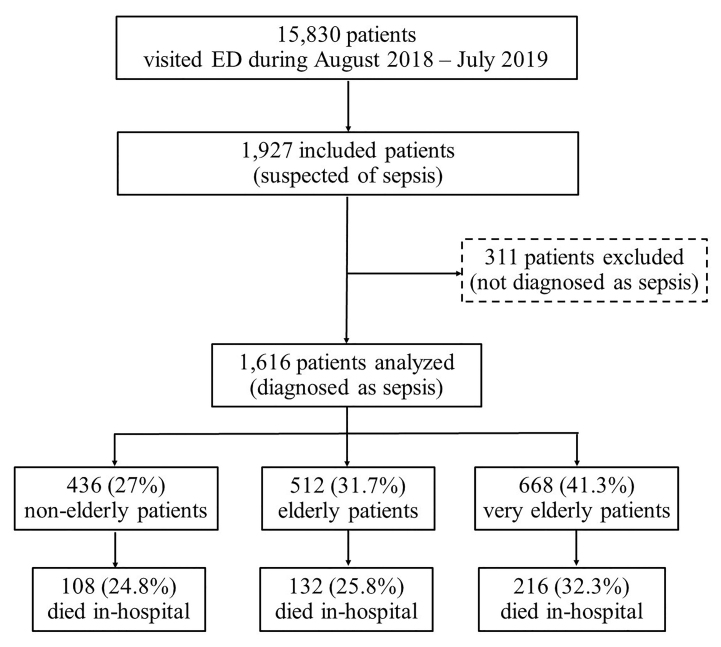
Flow diagram of the study participants. *ED*, emergency department.

A total of 15,830 patients visited the ED August 1, 2018–July 31, 2019. Of these, 1927 (12.2%) patients were in the ED due to suspected sepsis; 311 received empirical treatment and were not diagnosed as sepsis at discharge. There were no exclusions due to missing mortality status or incomplete early warning score values. Consequently, we analyzed 1616 patients. When stratified by age, 668 (41.3%) were in the very elderly group, 512 (31.7%) were in the elderly group, and 436 (27.0%) were non-elderly patients. The very elderly group had the highest mortality rate (32.3%), followed by the elderly (25.8%) and non-elderly (24.8%) groups (Figure 1).

Characteristics compared between the very elderly and the elderly patients are presented in Table 1. More of the very elderly group were female compared to the elderly group (*p*<0.0001). The very elderly group had significantly higher rates of underlying hypertension, debilitating neurologic diseases (ie, stroke, dementia), and bedridden and DNR status. Initial vital signs were similar between the two groups, except for a slightly higher systolic blood pressure in the very elderly group (*p* = 0.03). The very elderly group had significantly lower band form counts (*p* = 0.02), as well as a lower rate of positive hemoculture (*p* = 0.03). They also received fewer inotropic drugs (*p* = 0.02), and had fewer ICU admissions (*p* = 0.003) compared to the elderly group. When compared with other patients aged less than 80 years, the very elderly had significantly more underlying diseases. Moreover, more of them had sepsis due to respiratory and urinary tract infections. They also had higher mean systolic blood pressure and lower mean heart rate at presentation than younger patients (Table S1).

### Predictive Factors for In-Hospital Mortality

Table S2 presents characteristics of the very elderly comparing those who had and did not have in-hospital mortality. Table 1 reports results from univariate analyses of factors in predicting in-hospital mortality in the elderly and the very elderly group. In very elderly patients, factors chosen to be included in the multivariate model were age, underlying cancer, bedridden status, DNR status, recent hospital admission within the prior three months, suspected primary infection site, etiology of infection, SBP≤100, oxygen saturation, and GCS score. In the elderly, results were similar to the very elderly group, except that body temperature was a significant predictive factor for mortality.

From multivariate analyses, age (*p* = 0.03), DNR status (*p*<0.0001), history of recent admission (*p* = 0.02), respiratory tract infection (*p* = 0.03), SBP≤100 (*p* = 0.001), oxygen saturation (*p* = 0.002), and GCS score (*p*<0.0001) were independent factors associated with in-hospital mortality in the very elderly group. In the elderly group, factors that remained significant from multivariate analyses were DNR status (*p*<0.0001), body temperature (*p* = 0.006), and GCS score (*p*<0.0001) (Table 2). In the non-elderly group, factors associated with mortality were DNR status, oxygen saturation, and GCS score (Table S3). In the subgroup of patients without DNR status, the significant factor in predicting hospital mortality among all age group was GCS score. Body temperature remained a significant factor in the elderly group. In the very elderly patients, underlying hypertension, respiratory tract infection, and SBP≤100 were also predictive factors of mortality (Table S4).

### Performance of Early Warning Scores

SIRS, qSOFA and NEWS yielded higher AUROC in the very elderly compared to the elderly group (Table 3). AUROCs of SIRS and qSOFA increased with age. In the very elderly patients, qSOFA had the highest AUROC (0.60 [95% CI, 0.55–0.65]), followed by SIRS (0.55 [95% CI, 0.49–0.59]) and NEWS (0.54 [95% CI, 0.49–0.59]). NEWS≥5 had the highest sensitivity (89.8%) but lowest specificity (18.4%), whereas qSOFA≥2 yielded the highest specificity (71.0%) but lowest sensitivity (49.1%). Similar results were seen in the elderly group, except that SIRS≥2 could provide the highest sensitivity. Nonetheless, NEWS performed the best in the non-elderly group.

## DISCUSSION

This retrospective study found that the mortality rate of patients with sepsis increases with age. Patients aged 80 years and older had the highest mortality rate compared to patients aged 65–79 years and non-elderly patients. Moreover, age is found to be an independent predictive factor for in-hospital mortality in this very elderly group, but not in the other two younger cohorts. Initial vital signs may not be good predictors for mortality, unlike baseline mental status, which was shown to be predictive across all age groups. Furthermore, qSOFA was the best scoring system with the highest specificity and AUROC in predicting mortality in the elderly and the very elderly group.

The world population is experiencing an unprecedented demographic change. According to the 2019 world population prospects, the ratio of people aged over 65 will increase from 1/11 in 2019 to 1/6 in 2050. Additionally, people aged 80 or over will be tripled by the same time.^17^ These older adults are at increased risk of contracting infection due to declining physical and functional status. They are also at higher risk for developing sepsis and its adverse outcomes.^18^ In our study, we found that 73% of all patients with sepsis were aged 65 years or older, and the mortality rate increased with age. These findings were similar to previous studies conducted in ICUs^19^–^22^; however, the mortality rate in our study was relatively lower because it was conducted in the ED, not in the ICU where the severity and hence mortality rates of patients are usually higher. Besides, we found that over 40% of all patients were very elderly patients aged 80 years or older, which was higher than any previous studies conducted in ICU but similar to a study conducted in the ED.^23^ This might have been because ICU physicians usually consider ICU admissions for younger patients rather than very elderly patients with limited, life-sustaining treatment demand. We found that the ICU admission rate was significantly lower in the very elderly group compared to younger patients in our study.

Early identification of patients at high risk for developing adverse outcome from sepsis may aid clinicians to give appropriate treatment and may possibly lead to improved patient outcomes. For emergency physicians, vital signs and clinical characteristics at arrival are of utmost importance in order to early recognize patients at high risk. In fact, almost all components of early warning scores were based on this information. It is known that older patients usually present with atypical presentation and may not present with the abnormal vital signs usually seen in septic patients. Our study results showed supportive evidence.

First, the very elderly group had significantly lower heart rate compared to patients aged <80 years, and higher systolic blood pressure compared to both the elderly and all other patients aged <80 years. Although we found that SBP≤100 could significantly predict hospital mortality only in the very elderly group, this might have been because of the greater severity of disease in the very elderly compared to the other two groups. Second, we found that unlike in the elderly, body temperature was not an independent predictive factor of mortality in the very elderly. This was concordant with a previous report stating that the older the patients, the lower the body’s baseline temperature.^24^ Thus, fever may not be seen in geriatric patients with infection. However, we found that oxygen saturation is a significant factor in predicting mortality in the very elderly, but not in the elderly, which might have been due to the higher rate of respiratory tract infection in very elderly patients, similar to previous studies.^19^,^23^ Nonetheless, apart from all vital signs, the GCS may be a reliable tool to predict adverse outcome since it was a significant predictor across all age groups. This was also evident in the subgroup of patients without DNR status.

Of the commonly used early warning scores, qSOFA had the highest specificity and yielded the highest accuracy in predicting in-hospital mortality in the elderly and the very elderly, despite respiratory rate greater than 22 not being an independent predictive factor for mortality. This came as no surprise since qSOFA has always been known for its high specificity.^25^–^26^ It was proposed by Sepsis-3 as a tool to early identify patients with sepsis in the ED.^7^ However, recent studies in the general ED population have shown that newly-developed early warning scores, such as NEWS, may have better predictive performance than qSOFA and SIRS.^14^–^16^ We demonstrated similar findings in the non-elderly group, but not in the elderly and the very elderly groups. Interestingly, we found that AUROC of both qSOFA and SIRS increased in older patients, unlike NEWS. This might have been explained by the fewer number of components in qSOFA and SIRS.

Our data showed that many baseline variables in these scoring systems did not accurately predict mortality in older patients; therefore, the scoring systems with fewer variables could have yielded higher accuracy than those with more components. The qSOFA score only consists of three components, two of which are SBP≤100 and mental status that were found to be predictive of mortality. As a consequence, it could provide the highest accuracy in the very elderly patients. However, it is important to note that the prognostic accuracy performances based on sensitivity, specificity, and AUROC of all the early warning scores in this study were generally less robust than previous studies.^14^–^16^,^25^–^26^ This may have also been due to the advancing age and subsequently higher severity of patients’ baseline risk for mortality in this study population. A modification of the currently available scores or an “age” factor may be required to obtain better diagnostic and prognostic performances, as reported in previous studies.^27^–^28^ Nonetheless, further studies should still be conducted to derive and validate appropriate early warning scores for this particular population.

## LIMITATIONS

The study had several limitations. First, it was conducted in a single, tertiary, university hospital situated at the city center with moderate volume of visiting patients. This may limit the generalizability of the study findings. Second, we only included patients suspected of sepsis in the ED and not patients whom we did not suspect but later went on to be diagnosed with sepsis during hospital admission. This may have been reasonable since these patients could have had a hospital-acquired infection that occurred after ED disposition. However, we might have missed some patients with sepsis who presented with atypical presentation leading to non-sepsis-related diagnoses such as delirium. And although we included patients with DNR status who could have biased the study results in the primary analysis, we performed a subgroup analysis excluding them to obtain strong predictive factors that remained significant regardless of the patient’s palliative status. Nevertheless, some of the factors might have failed to meet statistical significance due to small sample sizes in the subgroup analyses.

Another limitation is that we used in-hospital mortality, which is all-cause mortality rather than sepsis-related mortality as the primary outcome. This might have overestimated the actual mortality due to sepsis since the elderly could have died from many other concurrent causes. Nonetheless, our mortality rate was similar to other previous studies in geriatric patients. Additionally, as per our clinical practice, we used the older definitions of hospital-acquired and healthcare-associated infection in the study. Finally, we did not have records of some essential factors in critical septic patients such as serum lactate, compliance with the sepsis bundle of care, or the severity of sepsis assessed by appropriate tools such as Sequential Organ Failure Assessment or Acute Physiology And Chronic Health Evaluation II (APACHE II) score. This was because of the retrospective nature of our study, which limited the completeness of laboratory data and the availability of variables needed for the score calculation. There might have also been other limitations associated with a retrospective study design such as potential selection bias.

## CONCLUSION

Very elderly patients with sepsis in the ED had higher in-hospital mortality than elderly and non-elderly patients. Factors associated with mortality in the very elderly were age, DNR status, history of recent admission, respiratory tract infection, SBP≤100, oxygen saturation, and GCS score. Factors associated with mortality in the elderly were DNR status, body temperature, and GCS score. qSOFA had the highest but only moderate accuracy in predicting in-hospital mortality in elderly and very elderly patients compared to SIRS and NEWS.

## Supplementary Information









## Figures and Tables

**Table 1:** Characteristics and factors predicting in-hospital mortality in elderly and very elderly patients.

Characteristics	Elderly (n=512)	OR (95%CI), P-value	Very elderly (n=668)	OR (95%CI), P-value	P-value of difference
Age	72.6+4.5	1.0 (0.9–1.00), 0.07	86.1+4.8	1.0 (1.0–1.1), 0.03	<0.0001
Gender (female)	238 (46.5)	1.1 (0.7–1.6), 0.78	386 (57.8)	0.9 (0.6–1.2), 0.48	<0.0001
Underlying conditions					
Diabetes mellitus	190 (37.1)	1.0 (0.6–1.4), 0.84	222 (33.2)	1.1 (0.8–1.5), 0.69	0.17
Hypertension	304 (59.4)	0.9 (0.6–1.3), 0.49	444 (66.5)	0.8 (0.6–1.2), 0.30	0.01
Dyslipidemia	187 (36.5)	1.2 (0.8–1.8), 0.43	257 (38.5)	1.1 (0.8–1.6), 0.51	0.49
CKD or ESRD	89 (17.4)	1.3 (0.8–2.2), 0.28	144 (21.6)	0.8 (0.6–1.2), 0.36	0.07
Coronary artery disease	63 (12.3)	0.9 (0.5–1.6), 0.70	110 (16.5)	0.8 (0.5–1.2), 0.21	0.05
Debilitating neurologic diseases	137 (26.8)	0.8 (0.5–1.3), 0.33	267 (40.0)	0.8 (0.6–1.1), 0.16	<0.0001
Cancer	136 (26.6)	2.4 (1.6–3.7), <0.0001	94 (14.1)	1.9 (1.2–2.9), 0.006	<0.0001
Bedridden status	306 (59.8)	1.9 (1.2–2.9), 0.004	563 (84.3)	1.6 (1.0–2.7), 0.04	<0.0001
Do-not-resuscitate status	192 (37.5)	4.8 (3.2–7.4), <0.0001	386 (57.8)	3.6 (2.5–5.2), <0.0001	<0.0001
Recent admission < 3 months	210 (41.0)	1.8 (1.2–2.7), 0.004	313 (46.9)	1.6 (1.2–2.2), 0.005	0.05
Suspected primary infection site					
Urinary tract	63 (12.3)	Ref, 0.09	100 (15.0)	Ref, 0.21	0.09
Respiratory tract	301 (58.8)	2.1 (1.0–4.4), 0.04	419 (62.7)	1.7 (1.0–2.8), 0.04	
Other known sites	50 (9.8)	0.8 (0.2–3.0), 0.69	42 (6.3)	1.3 (0.5–3.2), 0.62	
Unknown site	98 (19.1)	1.9 (0.9–4.2), 0.10	107 (16.0)	1.5 (0.8–2.6), 0.22	
Etiology of infection					
Community-acquired	278 (54.3)	Ref, 0.005	337 (50.4)	Ref, 0.04	0.08
Healthcare-associated	32 (6.3)	1.1 (0.4–2.6), 0.86	29 (4.3)	0.5 (0.2–1.3), 0.16	
Hospital-associated	202 (39.5)	2.0 (1.3–3.0), 0.001	302 (45.2)	1.4 (1.0–1.9), 0.06	
Vital signs and mental status at time of sepsis suspicion					
Body temperature (o^C^)	37.1 (36.8,37.9)	0.7 (0.6–0.9), 0.009	37.1 (36.8,37.9)	1.0 (1.0–1.0), 0.34	0.65
Respiratory rate (breaths/min)	30.9+8.4	1.0 (1.0–1.0), 0.67	31.2+8.2	1.0 (1.0–1.0), 0.95	0.47
Pulse rate (times/min)	102.9+41.9	1.4 (0.7–3.0), 0.33	97.8+42.3	1.5 (0.8–2.9), 0.21	0.07
Systolic blood pressure (mmHg)	125+36.1	1.6 (1.1–2.5), 0.03	129.9+40.5	1.8 (1.2–2.6), 0.004	0.03
Diastolic blood pressure (mmHg)	70.9+18.7	0.97 (0.95–0.99), 0.008	69.9+18.3	0.96 (0.94–0.98), <0.0001	0.34
Mean arterial pressure (mmHg)	89+23.1	0.8 (0.7–0.8), <0.0001	89.9+22.8	0.8 (0.7–0.8), <0.0001	0.49
Oxygen saturation (%)	94 (88,97)	0.7 (0.6–0.9), 0.009	94 (89,97)	1.0 (1.0–1.0), 0.34	0.48
Glasgow Coma Scale score	12.5+2.5	1.0 (1.0–1.0), 0.67	12.5+2.5	1.0 (1.0–1.0), 0.95	0.49
Laboratory results					
White blood cells (cells/mm^3^)	13,440+11,444	-	12,281+8,411	-	0.05
Band form (%)	3.3+15.1	-	1.8+5.8	-	0.02
Positive hemoculture	98 (19.1)	-	97 (14.5)	-	0.03
ED management					
Time to hemoculture (min)	28 (15,56)	-	30 (15,50)	-	0.34
Time to antibiotics (min)	107 (64,169)	-	99 (60,147)	-	0.23
Inotropic drugs	115 (22.5)	-	113 (16.9)	-	0.02
ED disposition					
ICU admission	33 (6.4)	-	19 (2.8)	-	0.003
Outcome					
Length of stay (days)	6 (2,11)	-	6 (2,11)	-	0.96
In-hospital mortality	132 (25.8)	-	216 (32.3)	-	0.01

Note: data presented as n (%), mean+ standard deviation or median (interquartile range).

*OR*, odds ratio; *CI*, confidence interval; *CKD*, chronic kidney disease; *ESRD*, end-stage renal disease; *Ref*, reference variable; *ED*, emergency department; *ICU*, intensive care unit; *mmHg*, millimeters of mercury; *mm^3^*, cubic millimeters.

**Table 2:** Multivariate analyses of factors associated with in-hospital mortality between elderly and very elderly patients.

Factors	Elderly (n=512)	P-value	Very elderly (n=668)	P-value
Age	1.0 (0.9–1.0)	0.43	1.0 (1.0–1.1)	0.03
Underlying conditions				
Cancer	1.4 (0.8–2.3)	0.26	1.4 (0.8–2.3)	0.20
Bedridden status	0.8 (0.5–1.4)	0.49	0.9 (0.5–1.6)	0.65
Do-not-resuscitate status	4.5 (2.6–7.6)	<0.0001	3.1 (2.0–4.8)	<0.0001
Recent admission < 3 months	0.6 (0.1–2.3)	0.42	3.5 (1.2–10.3)	0.02
Suspected primary infection site				
Urinary tract	Ref	0.20	Ref	0.13
Respiratory tract	1.6 (0.7–3.7)	0.25	1.9 (1.1–3.4)	0.03
Other known sites	0.6 (0.1–2.8)	0.53	1.1 (0.4–3.2)	0.88
Unknown site	1.0 (0.9–1.0)	0.43	1.5 (0.8–2.9)	0.26
Etiology of infection				
Community-acquired	Ref	0.35	Ref	0.11
Healthcare-associated	0.6 (0.2–1.6)	0.33	0.5 (0.2–1.3)	0.12
Hospital-associated	1.9 (0.5–8.0)	0.37	0.4 (0.1–1.1)	0.07
Vital signs and mental status at time of sepsis suspicion				
Body temperature (o^C^)	0.7 (0.5–0.9)	0.006	-	-
Systolic blood pressure<100 mmHg	1.3 (0.8–2.2)	0.36	2.2 (1.4–3.4)	0.001
Oxygen saturation (%)	1.0 (0.9–1.0)	0.13	0.97 (0.95–0.99)	0.002
Glasgow Coma Scale scores	0.7 (0.7–0.8)	<0.0001	0.8 (0.7–0.8)	<0.0001

Note: data presented as odds ratio (95% confidence interval).

*Ref*, reference variable; *mmHg*, millimeters mercury.

**Table 3:** Performances of early warning scores in predicting in-hospital mortality between non-elderly, elderly and very elderly patients.

Early warning scores	AUROC (95% CI)	Cut-point	Sensitivity	Specificity	LR+	LR−	PPV	NPV
SIRS								

Non-elderly	0.51 (0.45–0.57)	>2	88.1 (80.5–93.5)	14.4 (10.8–18.7)	1.0 (1.0–1.1)	0.8 (0.5–1.5)	25.2 (23.7–26.8)	78.7 (67.5–86.8)
Elderly	0.53 (0.47–0.58)		87.9 (81.1–92.9)	17.11 (13.5–21.3)	1.1 (1.0–1.2)	0.7 (0.4–1.2)	26.9 (25.4–28.5)	80.3 (70.9–87.1)
Very elderly	0.55 (0.49–0.59)		82.87 (77.2–87.6)	26.1 (22.1–30.4)	1.1 (1.0–1.2)	0.7 (0.5–0.9)	34.9 (33.1–36.8)	76.1 (69.6–81.6)

qSOFA								

Non-elderly	0.54 (0.48–0.61)	>2	40.4 (31.1–50.2)	68.5 (63.2–73.4)	1.3 (1.0–1.7)	0.9 (0.7–1.0)	29.5 (24.1–35.6)	77.8 (74.7–80.6)
Elderly	0.55 (0.49–0.61)		43.2 (34.6–52.1)	67.1 (62.1–71.8)	1.3 (1.0–1.7)	0.9 (0.7–1.0)	31.3 (26.4–36.8)	77.3 (74.3–80.0)
Very elderly	0.60 (0.55–0.65)		49.1 (42.2–55.9)	71.0 (66.6–75.2)	1.7 (1.4–2.0)	0.7 (0.6–0.8)	44.7 (39.9–49.7)	74.5 (71.7–77.1)

NEWS								

Non-elderly	0.55 (0.49–0.61)	>2	91.7 (84.9–96.2)	18.9 (14.9–23.6)	1.1 (1.1–1.2)	0.4 (0.2–0.9)	27.0 (25.5–28.6)	87.5 (78.3–93.2)
Elderly	0.52 (0.46–0.58)		87.1 (80.2–92.3)	16.8 (13.2–21.0)	1.1 (1.0–1.1)	0.8 (0.5–1.3)	26.7 (25.2–28.3)	79.0 (69.6–39.3)
Very elderly	0.54 (0.49–0.59)		89.8 (85.0–93.5)	18.4 (14.9–22.3)	1.1 (1.0–1.2)	0.6 (0.4–0.9)	34.5 (33.1–35.9)	79.1 (70.8–85.4)

Note: data presented as values (95%CI).

*AUROC*, area under receiver operating characteristic curve; *CI*, confidence interval; *LR+*, positive likelihood ratio; *LR−*, negative likelihood ratio; *PPV*, positive predictive value; *NPV*, negative predictive value; *SIRS*, systemic inflammatory response score; *qSOFA*, quick sequential organ failure assessment score; *NEWS*, National Early Warning Score.
