# Supplementary material for: Predictors of Mortality in Elderly and Very Elderly Emergency Patients with Sepsis: A Retrospective Study
Source: West J Emerg Med. 2020 Oct 6;21(6):210–8. doi: 10.5811/westjem.2020.7.47405 (PMC7673873; doi:10.5811/westjem.2020.7.47405)
Supplement: Supplementary file 1 [file wjem-21-210-s001.docx]

**Table S1.** Characteristics compared between very elderly patients and all other younger patients.

| **Characteristics** | **All**  **(n=1,616)** | **Very elderly**  **(n=668)** | **Others**  **(n=948)** | **P-value** |
| --- | --- | --- | --- | --- |
| Sex(female) | 826 (51.1) | 386 (57.8) | 439 (46.3) | <0.0001 |
| **Underlying conditions** |  |  |  |  |
| Diabetes mellitus | 511 (31.6) | 222 (33.2) | 289 (30.5) | 0.24 |
| Hypertension | 864 (53.5) | 424 (63.5) | 440 (46.4) | <0.0001 |
| Dyslipidemia | 524 (32.4) | 257 (38.5) | 266 (28.1) | <0.0001 |
| CKD or ESRD | 292 (18.1) | 144 (21.6) | 149 (15.7) | 0.003 |
| Coronary artery disease | 205 (12.7) | 110 (16.5) | 95 (10.0) | <0.0001 |
| Debilitating neurologic diseases | 448 (27.7) | 267 (40.0) | 182 (19.2) | <0.0001 |
| Cancer | 367 (22.7) | 94 (14.1) | 273 (28.8) | <0.0001 |
| Bedridden status | 1,089 (67.4) | 563 (84.3) | 528 (55.7) | <0.0001 |
| Do-not-resuscitate status | 716 (44.3) | 386 (74.8) | 331 (34.9) | <0.0001 |
| Recent admission <3 months | 726 (44.9) | 313 (46.9) | 413 (43.6) | 0.31 |
| **Suspected primary infection site** |  |  |  |  |
| Urinary tract | 197 (12.2) | 100 (15.0) | 98 (10.3) | 0.002 |
| Respiratory tract | 978 (60.5) | 419 (62.7) | 559 (59.0) |  |
| Other known sites | 139 (8.6) | 42 (6.3) | 97 (10.2) |  |
| Unknown site | 302 (18.7) | 107 (16.0) | 194 (20.5) |  |
| **Etiology of infection** |  |  |  |  |
| Community-acquired | 827 (51.2) | 337 (50.4) | 490 (51.7) | 0.15 |
| Healthcare-associated | 89 (5.5) | 29 (4.3) | 60 (6.3) |  |
| Hospital-associated | 700 (43.3) | 302 (45.2) | 398 (42.0) |  |
| **Vital signs and mental status at time of sepsis suspicion** | | | | |
| Body temperature (^o^C) | 37.1 (36.8,38.0) | 37.1 (36.8,37.9) | 37.1 (36.8,38.0) | 0.51 |
| Respiratory rate (breaths/min) | 31.1+8.6 | 31.2+8.2 | 31.1+8.8 | 0.72 |
| Pulse rate (times/min) | 102.6+38.4 | 97.8+42.3 | 105.9+35.1 | <0.0001 |
| Systolic blood pressure (mmHg) | 125.4+37.3 | 129.9+40.6 | 122.1+34.4 | <0.0001 |
| Diastolic blood pressure (mmHg) | 71.3+26.7 | 69.9+18.4 | 72.2+31.2 | 0.09 |
| Mean arterial pressure (mmHg) | 89.3+26.2 | 89.9+22.9 | 88.9+28.2 | 0.42 |
| Oxygen saturation (%) | 95 (89,97) | 94 (89,97) | 95 (89,97) | 0.60 |
| Glasgow coma scale score | 12.5+2.5 | 12.4+2.5 | 12.5+2.5 | 0.50 |
| **Laboratory results** |  |  |  |  |
| White blood cells (cells/mm^3^) | 12,822.5+9,514.8 | 12,281.2+8,417.9 | 13,201.5+10,200.2 | 0.06 |
| Band form (%) | 2.5+10 | 1.8+5.8 | 3+12.1 | 0.005 |
| Positive hemoculture | 275 (17.0) | 97 (14.5) | 178 (18.8) | 0.03 |
| **ED management** |  |  |  |  |
| Time to hemoculture (min) | 30 (16,55) | 30 (15,50) | 29 (16,60) | 0.22 |
| Time to antibiotics (min) | 103 (64,160) | 98 (60,147) | 108 (64,169) | 0.09 |
| Inotropic drugs | 339 (21.0) | 113 (16.9) | 226 (23.8) | 0.001 |
| **ED disposition** |  |  |  |  |
| ICU admission | 82 (5.1) | 19 (2.8) | 63 (6.6) | 0.001 |
| **Outcome** |  |  |  |  |
| Length of stay (days) | 6 (2,11) | 6 (2,11) | 6 (2,11) | 0.74 |
| In-hospital mortality | 456 (28.2) | 216 (32.3) | 240 (25.3) | 0.002 |

Note: data presented as n (%), mean+SD or median (IQR) Abbreviations: CKD, chronic kidney disease; ESRD, end-stage renal disease; ED, emergency department; ICU, intensive care unit.
