# Supplementary material for: Predictors of Mortality in Elderly and Very Elderly Emergency Patients with Sepsis: A Retrospective Study
Source: West J Emerg Med. 2020 Oct 6;21(6):210–8. doi: 10.5811/westjem.2020.7.47405 (PMC7673873; doi:10.5811/westjem.2020.7.47405)
Supplement: Supplementary file 2 [file wjem-21-210-s002.docx]

**Table S2.** Characteristics of very elderly patients compared between patients who had and did not have hospital mortality.

| **Characteristics** | **All**  **(n=668)** | **Dead**  **(n=216)** | **Alive**  **(n=452)** | **P-value** |
| --- | --- | --- | --- | --- |
| Sex (female) | 386 (57.8) | 129 (59.7) | 257 (56.9) | 0.48 |
| **Underlying conditions** |  |  |  |  |
| Diabetes mellitus | 222 (33.2) | 74 (34.3) | 148 (32.7) | 0.69 |
| Hypertension | 424 (63.5) | 131 (60.6) | 293 (64.8) | 0.30 |
| Dyslipidemia | 257 (38.5) | 87 (40.3) | 170 (37.6) | 0.51 |
| CKD or ESRD | 144 (21.6) | 42 (19.4) | 102 (22.6) | 0.36 |
| Coronary artery disease | 110 (16.5) | 30 (13.9) | 80 (17.7) | 0.21 |
| Debilitating neurologic diseases | 267 (40.0) | 78 (36.1) | 189 (41.8) | 0.16 |
| Cancer | 94 (14.1) | 42 (19.4) | 52 (11.5) | 0.10 |
| Bedridden status | 563 (84.3) | 191 (88.4) | 372 (82.3) | 0.04 |
| Do-not-resuscitate status | 386 (57.8) | 167 (77.3) | 219 (48.5) | <0.0001 |
| Recent admission <3 months | 313 (46.9) | 118 (54.6) | 195 (43.1) | 0.01 |
| **Suspected primary infection site** |  |  |  |  |
| Urinary tract | 100 (15.0) | 24 (11.1) | 76 (16.8) | 0.03 |
| Respiratory tract | 419 (62.7) | 146 (67.6) | 273 (60.4) |  |
| Other known sites | 42 (6.3) | 8 (3.7) | 34 (7.5) |  |
| Unknown site | 107 (16.0) | 38 (17.6) | 69 (15.3) |  |
| **Etiology of infection** |  |  |  |  |
| Community-acquired | 337 (50.4) | 100 (46.3) | 237 (52.4) | 0.03 |
| Healthcare-associated | 29 (4.3) | 5 (2.3) | 24 (5.3) |  |
| Hospital-associated | 302 (45.2) | 111 (51.4) | 191 (42.3) |  |
| **Vital signs and mental status at time of sepsis suspicion** | | | | |
| Body temperature (^o^C) | 37.1 (36.8,37.9) | 37.0 (36.7,37.6) | 37.1 (36.8,38.0) | 0.34 |
| Respiratory rate (breaths/min) | 31.2+8.2 | 32.3+7 | 30.7+8.7 | 0.02 |
| Pulse rate (times/min) | 97.8+42.3 | 97.9+26.8 | 97.8+48 | 0.95 |
| Systolic blood pressure (mmHg) | 130+40.6 | 127.2+52.5 | 131.3+33.4 | 0.22 |
| Diastolic blood pressure (mmHg) | 69.9+18.4 | 68.2+19.7 | 70.8+17.7 | 0.09 |
| Mean arterial pressure (mmHg) | 89.9+22.9 | 87.8+25.7 | 90.9+21.3 | 0.10 |
| Oxygen saturation (%) | 94 (89,97) | 92 (85,97) | 95 (90,97) | 0.001 |
| Glasgow coma scale score | 12.4+2.5 | 11.3+2.9 | 12.9+2.1 | <0.0001 |
| **Laboratory results** |  |  |  |  |
| White blood cells (cells/mm^3^) | 12,281.2+8,417.9 | 13,378.1+9,464.7 | 11,757.0+7,825.9 | 0.03 |
| Band form (%) | 1.8+5.8 | 2.4+7.3 | 1.5+4.9 | 0.07 |
| Positive hemoculture | 97 (14.5) | 41 (19.0) | 56 (12.4) | 0.02 |
| **ED management** |  |  |  |  |
| Time to hemoculture (min) | 30 (15,50) | 28 (14,40) | 31 (17,56) | 0.78 |
| Time to antibiotics (min) | 99 (60,147) | 97 (58,145) | 100 (63,149) | 0.62 |
| Inotropic drugs | 113 (16.9) | 58 (26.9) | 55 (12.2) | <0.0001 |
| **ED disposition** |  |  |  |  |
| ICU admission | 19 (2.8) | 10 (4.6) | 9 (2) | <0.0001 |
| **Outcome** |  |  |  |  |
| Length of stay (days) | 6 (2,11) | 4 (2,11) | 6 (2,11) | 0.01 |

Note: data presented as n (%), mean+SD or median (IQR) Abbreviations: CKD, chronic kidney disease; ESRD, end-stage renal disease; ED, emergency department; ICU, intensive care unit.
