# Supplementary material for: Predictors of Mortality in Elderly and Very Elderly Emergency Patients with Sepsis: A Retrospective Study
Source: West J Emerg Med. 2020 Oct 6;21(6):210–8. doi: 10.5811/westjem.2020.7.47405 (PMC7673873; doi:10.5811/westjem.2020.7.47405)
Supplement: Supplementary file 3 [file wjem-21-210-s003.docx]

**Table S3.** Univariate and multivariate analyses of factors associated with in-hospital mortality in non-elderly patients.

| **Factors** | | **Age<65 years**  **(n=436)** | **OR (95%CI)** | **P-value** | **Adjusted OR (95% CI)** | **P-value** |
| --- | --- | --- | --- | --- | --- | --- |
| Age | 52.2+11.3 | | 1.0 (1.0-1.0) | 0.44 | - | - |
| Sex (female) | 202 (46.3) | | 0.8 (0.6-1.0) | 0.15 | - | - |
| **Underlying conditions** |  | |  |  |  |  |
| Diabetes mellitus | 100 (22.9) | | 0.8 (0.5-1.4) | 0.45 | - | - |
| Hypertension | 136 (31.2) | | 0.8 (0.5-1.2) | 0.28 | - | - |
| Dyslipidemia | 80 (18.3) | | 1.1 (0.6-1.9) | 0.77 | - | - |
| CKD or ESRD | 60 (13.8) | | 0.8 (0.4-1.6) | 0.51 | - | - |
| Coronary artery disease | 32 (7.3) | | 0.7 (0.3-1.6) | 0.37 | - | - |
| Debilitating neurologic diseases | 45 (10.3) | | 1.2 (0.6-2.4) | 0.55 | - | - |
| Cancer | 137 (31.4) | | 3.2 (2.0-5.0) | <0.0001 | 1.6 (0.9-2.9) | 0.10 |
| Bedridden status | 222 (50.9) | | 2.3 (1.5-3.6) | <0.0001 | 1.0 (0.5-1.9) | 0.96 |
| Do-not-resuscitate status | 139 (31.9) | | 6.6 (4.1-10.6) | <0.0001 | 5.1 (2.8-9.4) | <0.0001 |
| Recent admission <3 months | 203 (46.6) | | 1.5 (1.0-2.3) | 0.08 | 0.5 (0.2-1.7) | 0.21 |
| **Suspected primary infection site** |  | |  |  |  |  |
| Urinary tract | 34 (7.8) | | Ref | 0.50 | - | - |
| Respiratory tract | 258 (59.2) | | 0.8 (0.4-1.7) | 0.53 | - | - |
| Other known sites | 47 (10.8) | | 0.4 (0.1-1.5) | 0.18 | - | - |
| Unknown site | 97 (22.2) | | 1.0 (0.4-2.3) | 0.97 | - | - |
| **Etiology of infection** |  | |  |  |  |  |
| Community-acquired | 212 (48.6) | | Ref | 0.14 | Ref | 0.29 |
| Healthcare-associated | 28 (6.4) | | 1.0 (0.4-2.7) | 0.95 | 0.7 (0.2-2.1) | 0.53 |
| Hospital-associated | 196 (45.0) | | 1.6 (1.00-2.4) | 0.05 | 2.1 (0.6-7.2) | 0.23 |
| **Vital signs and mental status at time of sepsis suspicion** | | | |  |  |  |
| Body temperature (^o^C) | 37.1 (36.8,38.3) | | 0.9 (0.7-1.0) | 0.12 | - | - |
| Pulse rate (times/min) | 109.5+24.5 | | 1.0 (1.0-1.0) | 0.02 | 1.0 (1.0-1.0) | 0.54 |
| Respiratory rate>22 breaths/mins | 391(88.5) | | 1.2 (0.6-2.5) | 0.59 | - | - |
| Systolic blood pressure<100 mmHg | 141(31.9) | | 0.9 (0.6-1.5) | 0.68 | - | - |
| Oxygen saturation (%) | 95(90,98) | | 0.97 (0.95-0.99) | 0.004 | 0.97 (0.95-0.99) | 0.026 |
| Glasgow coma scale score | 12.5+2.6 | | 0.8 (0.7-0.8) | <0.0001 | 0.8 (0.7-0.9) | <0.0001 |

Note: data presented as n(%), mean+SD, median(IQR). Abbreviations: OR, odds ratio; CI, confidence interval; CKD, chronic kidney disease; ESRD, end stage renal disease; Ref, reference variable.
