# Supplementary material for: Predictors of Mortality in Elderly and Very Elderly Emergency Patients with Sepsis: A Retrospective Study
Source: West J Emerg Med. 2020 Oct 6;21(6):210–8. doi: 10.5811/westjem.2020.7.47405 (PMC7673873; doi:10.5811/westjem.2020.7.47405)
Supplement: Supplementary file 4 [file wjem-21-210-s004.docx]

| **Factors** | **Non-elderly (n=301)** | | **Elderly (n=320)** | | | **Very elderly (n=282)** | |
| --- | --- | --- | --- | --- | --- | --- | --- |
|  | **OR (95%CI):**  **P-value** | **Adjusted OR (95% CI): P-value** | **OR (95%CI):**  **P-value** | | **Adjusted OR (95% CI): P-value** | **OR (95%CI):**  **P-value** | **Adjusted OR (95% CI): P-value** |
| Age | 1.0 (1.0-1.0); 0.11 | - | 1.0 (0.9-1.1); 0.87 | | - | 1.0 (1.0-1.1); 0.20 | - |
| Sex (female) | 1.9 (0.9-3.8); 0.08 | - | 1.3 (0.7-2.5); 0.41 | | - | 1.0 (0.5-1.8); 0.93 | - |
| **Underlying conditions** |  |  |  | |  |  |  |
| Diabetes mellitus | 0.5 (0.2-1.2); 0.14 | - | 0.9 (0.5-1.8); 0.82 | | - | 1.4 (0.7-2.6); 0.34 | - |
| Hypertension | 0.5 (0.2-1.1); 0.07 | - | 0.7 (0.4-1.2); 0.19 | | - | 0.5 (0.3-0.9); 0.03 | 0.4 (0.2-0.9); 0.02 |
| Dyslipidemia | 0.8 (0.3-2.0); 0.62 | - | 0.8 (0.4-1.5); 0.46 | | - | 1.0 (0.5-1.8); 0.94 | - |
| CKD or ESRD | 0.6 (0.2-1.7); 0.33 | - | 2.1 (1.1-4.2); 0.03 | | 2.2 (1.0-4.8); 0.04 | 0.6 (0.3-1.4); 0.25 | - |
| Coronary artery disease | 0.5 (0.1-2.4); 0.41 | - | 1.1 (0.4-2.5); 0.91 | | - | 0.8 (0.3-1.8); 0.54 | - |
| Debilitating neurologic diseases | 1.1 (0.3-3.8); 0.92 | - | 0.5 (0.2-1.2); 0.15 | | - | 0.5 (0.2-1.0); 0.06 | 0.5 (0.2-1.1); 0.08 |
| Cancer | 1.8 (0.8-4.0); 0.14 | - | 1.6 (0.7-3.5); 0.28 | | - | 1.8 (0.6-5.2); 0.29 | - |
| Bedridden status | 1.3 (0.6-2.5); 0.50 | - | 1.2 (0.6-2.2); 0.64 | | - | 1.0 (0.5-2.0); 0.98 | - |
| Recent admission <3 months | 0.2 (0.1-0.4); <0.0001 | 0.2 (0.1-0.5); 0.001 | 0.3 (0.1-0.6); 0.003 | | 0.3 (0.1-0.7); 0.01 | 0.4 (0.1-1.3); 0.14 | - |
| **Suspected primary infection site** | |  |  | |  |  |  |
| Urinary tract | Ref; 0.06 | - | Ref; 0.31 | | - | Ref; 0.11 | Ref; 0.06 |
| Respiratory tract | 0.9 (0.2-4.3); 0.92 | - | 3.7 (0.8-16.2); 0.08 | | - | 5.5 (1.3-24.0); 0.02 | 7.5 (1.5-38.6); 0.02 |
| Other known sites | 0.7 (0.1-5.8); 0.77 | - | 0.0 (0.0-0.0); 1.0 | | - | 5.1 (0.8-34.3); 0.09 | 5.5 (0.7-45.4); 0.12 |
| Unknown site | 2.4 (0.5-11.2); 0.28 | - | 4.4 (1.0-20.1); 0.06 | | - | 3.5 (0.7-17.4); 0.13 | 3.6 (0.6-21.1); 0.16 |
| **Etiology of infection** |  |  |  | |  |  |  |
| Community-acquired | Ref; 0.63 | - | Ref; 0.45 | | - | Ref; 0.90 | - |
| Healthcare-associated | 0.6 (0.1-5.0); 0.65 | - | 0.5 (0.1-4.0); 0.51 | | - | 0.0 (0.0-0.0); 1.0 | - |
| Hospital-associated | 1.3 (0.7-2.6); 0.45 | - | 1.4 (0.7-2.7); 0.32 | | - | 0.9 (0.4-1.6); 0.64 | - |
| **Vital signs and mental status at time of sepsis suspicion** | | |  |  | |  |  |
| Body temperature (^o^C) | 0.8 (0.7-1.0); 0.10 | - | 0.6 (0.4-0.9); 0.04 | | 0.4 (0.3-0.7); 0.001 | 1.1 (0.8-1.5); 0.64 | - |
| Pulse rate (times/min) | 1.0 (1.0-1.0); 0.09 | - | 1.0 (1.0-1.0); 0.26 | | - | 1.0 (1.0-1.0); 0.78 | - |
| Respiratory rate>22 breaths/mins | 1.3 (0.4-3.8); 0.68 | - | 1.0 (0.4-2.8); 0.93 | | - | 1.6 (0.5-5.6); 0.46 | - |
| Systolic blood pressure<100 mmHg | 0.8 (0.4-1.8); 0.63 | - | 1.9 (1.0-3.6); 0.06 | | - | 2.3 (1.1-4.6); 0.03 | 2.7 (1.2-6.3); 0.02 |
| Oxygen saturation (%) | 0.98 (0.96-1.0); 0.19 | - | 1.0 (0.9-1.0); 0.10 | | - | 0.96 (0.92-0.99); 0.03 | 1.0 (0.9-1.0); 0.07 |
| Glasgow coma scale score | 0.7 (0.6-0.8); <0.0001 | 0.7 (0.6-0.8); <0.0001 | 0.7 (0.6-0.8); <0.0001 | | 0.6 (0.5-0.7); <0.0001 | 0.8 (0.7-0.9); <0.0001 | 0.7 (0.7-0.8); <0.0001 |

**Table S4.** Univariate and multivariate analyses of factors associated with in-hospital mortality in subgroup of patients without do-not-resuscitate order.

Note: data presented as OR(95%CI), p-value. Abbreviations: OR, odds ratio; CI, confidence interval; CKD, chronic kidney disease; ESRD, end stage renal disease; Ref, reference variable.
